# Behavior Change Techniques Included in Reports of Social Media Interventions for Promoting Health Behaviors in Adults: Content Analysis Within a Systematic Review

**DOI:** 10.2196/16002

**Published:** 2020-06-11

**Authors:** Rosiane Simeon, Omar Dewidar, Jessica Trawin, Stephanie Duench, Heather Manson, Jordi Pardo Pardo, Jennifer Petkovic, Janet Hatcher Roberts, Peter Tugwell, Manosila Yoganathan, Justin Presseau, Vivian Welch

**Affiliations:** 1 Bruyère Research Institute University of Ottawa Ottawa, ON Canada; 2 Faculty of Medicine School of Epidemiology and Public Health University of Ottawa Ottawa, ON Canada; 3 Department of Population Medicine University of Guelph Guelph, ON Canada; 4 Dalla Lana School of Public Health University of Toronto Toronto, ON Canada; 5 Cochrane Musculoskeletal Group Ottawa Hospital Research Institute Ottawa, ON Canada; 6 Department of Medicine Faculty of Medicine University of Ottawa Ottawa, ON Canada; 7 Clinical Epidemiology Program Ottawa Health Research Institute Ottawa, ON Canada; 8 School of Psychology Faculty of Social Sciences University of Ottawa Ottawa, ON Canada

**Keywords:** health behavior, taxonomy, social media, health promotion, public health

## Abstract

**Background:**

Social media are an increasingly commonly used platform for delivering health promotion interventions. Although recent research has focused on the effectiveness of social media interventions for health promotion, very little is known about the optimal content within such interventions, and the active ingredients to promote health behavior change using social media are not clear. Identifying which behavior change techniques (BCTs) are reported may help to clarify the content of interventions using a generalizable terminology that may facilitate future intervention development.

**Objective:**

This study aimed to identify which BCTs are reported in social media interventions for promoting health behavior change in adults.

**Methods:**

We included 71 studies conducted with adult participants (aged ≥18 years) and for which social media intervention was considered interactive in a Cochrane review of the effectiveness of such interventions. We developed a coding manual informed by the Behavior Change Technique Taxonomy version 1 (BCTTv1) to identify BCTs in the included studies. We identified BCTs in all study arms (including control) and described BCTs in the group and self-directed components of studies. We characterized the dose of delivery for each BCT by low and high intensity. We used descriptive analyses to characterize the reported BCTs.

**Results:**

Our data consisted of 71 studies published from 2001 to 2017, mainly conducted in high-income countries (n=65). Most studies (n=31) used tailored, interactive websites to deliver the intervention; Facebook was the most used mainstream platform. In developing our coding manual, we adapted some BCTTv1 instructions to better capture unique nuances of how BCTs were operationalized in social media with respect to likes, retweets, smiles, congratulations, and badges. Social support (unspecified), instruction on how to perform the behavior, and credible source were most frequently identified BCTs in intervention arms of studies and group-delivery settings, whereas instruction on how to perform the behavior was most commonly applied in self-directed components of studies, control arms, and individual participant settings. Instruction on how to perform the behavior was also the most frequently reported BCT in both intervention and control arms simultaneously. Instruction on how to perform the behavior, social support (unspecified), self-monitoring of behavior, information about health consequences, and credible source were identified in the top 5 BCTs delivered with the highest intensity.

**Conclusions:**

This study within a review provides a detailed description of the BCTs and their dose to promote behavior change in web-based, interactive social media interventions. Clarifying active ingredients in social media interventions and the intensity of their delivery may help to develop future interventions that can more clearly build upon the existing evidence.

## Introduction

### Background

With more than 4 billion users worldwide and a projected yearly growth of 7% [1], the internet has become a central means of delivering health promotion interventions [2,3]. In particular, the advent and widespread use of social media have fostered a novel setting in which to deliver public health and health equity–oriented interventions [2,3]. These interventions greatly differ in their health aims, including HIV testing [4], mental health [5], physical activity [6], smoking cessation [7], and vaccination [8]. Social media can be characterized as consisting of “activities, practices, and behaviors among communities of people who gather online to share information, knowledge, and opinions using conversational media...that make it possible to create and easily transmit content in the form of words, pictures, videos, and audios” [9]. A primary goal of these social media interactions in health promotion is to change health behavior.

Interactions among participants in web-enabled interventions can lead to better health outcomes [10,11]. The past decade of research suggests that social media interventions may be effective in promoting health behavior change [12,13]. Earlier research has highlighted the need to improve the assessment of the design, implementation, and effectiveness of health promotion interventions delivered through social network technologies [14]. The findings from a recent review have revealed the diversity of social media features used in health-focused interventions delivered through the internet [15]. These features support several functions, including identity representation, peer grouping, and web-based social networking [15]. However, it is currently unclear which specific techniques are delivered within social media interventions, that is, which *active ingredients* are leveraged to promote behavior change in this new medium [11,12]. The active ingredient of an intervention that aims to change behavior can be defined as a behavior change technique (BCT), which consists of “an observable, replicable, and irreducible component of an intervention designed to alter or redirect causal processes that regulate behaviour” [16]. It is, therefore, important to describe the content of interventions delivered in social media and unpack their *active ingredients* to determine (1) what is actually being delivered in both intervention and control groups, (2) gaps and opportunities to consider integrating underrepresented BCTs, and (3) if this delivery mode of social media presents new BCTs or novel ways of operationalizing BCTs. Using a shared language to describe the intervention content, including which BCTs are used and how they are employed within an intervention, can help elucidate the development of future effective interventions.

Identification of BCTs using an agreed taxonomy ensures consistent comparison of techniques across studies, building toward a cumulative evidence base for informing terminology and for chartering the active ingredients of social media interventions. The Behavior Change Technique Taxonomy version 1 (BCTTv1) was developed to identify such active ingredients and to characterize the components of strategies that aim to change behavior [16-18]. The BCTTv1 with its 93 individual BCTs grouped into 16 categories [16] provides a standardized approach to classifying the content of health promotion interventions that involve behavior change. The taxonomy has been used extensively to clarify the active ingredients of health behavior change interventions in systematic reviews across several settings [19-21]. In addition, the effectiveness of health behavior change interventions in other areas has been shown to be associated with the inclusion of particular BCTs [22-24]. The findings from a systematic review that assessed the behavioral mechanisms of social media interventions on adolescent diet suggested that BCTs such as goal setting and self-monitoring of behavior may have a positive impact on changing dietary habits in adolescents [25]. However, the authors highlight the need to improve the description of BCTs delivered in social media interventions [25].

### Objectives

In this study within a review (SWAR) [26], we aimed to gain a better understanding of the active ingredients of social media interventions by identifying and describing BCTs and assessing the applicability of the BCT framework to studies considered for a Cochrane review of the effectiveness of interactive social media interventions [27].

## Methods

### Identification of Studies

The details of the systematic review methods, including the search strategy, are available in our published protocol [27]. We searched the following major electronic databases: Cochrane Central Register of Controlled Trials (CENTRAL), Medical Literature Analysis and Retrieval System Online, or MEDLARS Online (MEDLINE), EMBASE, Cumulative Index to Nursing and Allied Health Literature (CINAHL), and PsycINFO. We also conducted a focused search for unpublished studies or reports within Google Scholar and Web of Science. The search was also extended to websites of public health governmental and nongovernmental organizations, such as the Public Health Agency of Canada, the World Health Organization (WHO), and international development agencies such as the Asian Development Bank and the Inter‐American Development Bank. Clinical trial registries and the WHO International Clinical Trials Registry Platform were also searched for relevant studies. We also searched the reference lists of the included studies. Multimedia Appendix 1 provides a sample of the search strategy and results from MEDLINE. In brief, we included studies focused on individuals from the general population who were aged ≥18 years with no limitations on their health status. We only included social media interventions that allowed two-way communication and interaction between and among participants. Some examples consisted of interventions supported through major (as of 2019) social media outlets, such as Facebook, Instagram, Twitter, and YouTube. However, interventions implemented through these platforms had to allow peer interaction to be considered in this review.

Study comparators included usual care, no intervention, or an active comparison (eg, one type of social media compared with another). The primary outcomes included validated measures of health-related behaviors, physical health, well-being, and psychological health. Studies were not excluded based on the outcome. The literature search was limited to studies that were published between January 2001 and March 2017 as most of the commonly used social media platforms were developed in 2001 or later (eg, Facebook and Twitter), and our overview showed no earlier studies using these (currently) widely used social media platforms [3]. The types of included studies consisted of randomized controlled trials (RCTs), controlled before-and-after studies, interrupted time series, and RCTs with stepped-wedge designs.

We excluded interventions that used one-way communication or one-to-one communication between a user and practitioner. For example, we excluded studies that assessed mobile health apps (eg, apps that track clinical information with contact between an individual and their health care provider) and studies with content that is transmitted unidirectionally (eg, text message reminder interventions in which the recipient is unable to reply and podcasts in which health information is provided with no opportunity for two-way communication) or which only allows for comments without sharing functionality, such as blogs. We also excluded studies that assessed web-based interventions that are based on the exchange between a single care provider and an individual participant, such as web-based cognitive behavioral therapy. Advertisements on social media (eg, Facebook) without interactive functionality and virtual gaming interventions were also excluded.

### Coding and Analysis Strategies

For each eligible study, 2 trained coders (JT and RS) used BCTTv1 as a basis for coding BCTs. Coders completed BCTTv1 training and met the requirements for accurate and reliable coding. Following training, coders calibrated their coding by independently coding 3 included studies to resolve any initial discrepancies and to inform the development of a contextualized coding manual to supplement the BCTTv1. This was an iterative process to adapt the instructions if needed, which were applied to previously coded and future studies for testing. We developed a coding manual and identified any BCTs that were unique to social media. Multimedia Appendix 2 provides more details on the coding and analysis strategies related to this review.

We also distinguished between low- and high-intensity use to allow a balanced characterization of BCTs in interventions. In this review, we defined low-intensity BCT delivery as a given BCT delivered using one modality at one time. We coded high-intensity BCT delivery as a given BCT delivered more than once using the same or different modalities. Multimedia Appendix 2 provides examples of BCT statements coded as high or low intensity.

The titles of the included studies were entered in an Excel sheet, which contained all 93 BCTs organized under their respective categories. Our coding process captured the distribution of BCTs in interactive social media and self-directed components of the included studies. Interactive social media were defined as two-way communication and interaction between and among participants. Self-directed activities consisted of those carried out by participants outside of social media, such as tracking steps in a diary or using a pedometer. BCTs were extracted in both intervention and control arms in reported papers, appendices, websites, and other references indicated by the authors. We also recorded BCTs applied to participants in a group and individual participants. An interrater reliability score of kappa value 0.60 was used before starting to code [25]. Reviewers convened weekly to reconcile coding discrepancies. A third researcher (J Presseau) was consulted when discrepancies could not be resolved. Frequency counts were performed separately to quantify the categories of BCTs recorded and the intensity with which these were applied. A descriptive analysis was conducted on the identification and application of BCTs in interactive social media interventions.

## Results

### Characteristics of the Included Studies

Studies were identified using the search strategy specified in the systematic review protocol [27]. Our team screened more than 25,000 records and assessed 282 full-text studies for eligibility criteria. After applying the eligibility criteria, 71 studies were retained eligible for analysis. The majority of the studies consisted of RCTs (60 studies). As shown in Table 1, the studies reviewed were mainly conducted in high-income countries, which hosted 92% (65/71) of the interventions. Only 5 interventions were conducted in upper–middle-income countries. There was only 1 study identified in lower–middle-income countries.

**Table 1:** Summary of characteristics of included studies (N=71).

Study characteristics		Value, n (%)
**Country**		
	High-income countries	65 (92)
	Upper–middle-income countries	5 (7)
	Lower–middle-income countries	1 (1)
**Study design**		
	RCT^a^	60 (85)
	Non-RCT	11 (15)
**Types of social media**		
	Tailored interactive website only	31 (44)
	Facebook only	23 (32)
	Tailored interactive website and other social media components (Facebook and interactive apps)	13 (18)
	Twitter or WhatsApp or WeChat	3 (4)
	Twitter and other social media components	1 (1)
**Types of outcomes^b^**		
	Health-related behaviors	40 (56)
	Physical health	25 (35)
	Well-being	11 (15)
	Psychological health	8 (11)

^a^RCT: randomized controlled trial.

^b^More than one outcome per study.

Interactive social media attributes were identified in both the experimental and control arms of the included studies. In studies with active comparators, study arms were distinguished by changing the intensity of BCTs delivered [28,29], varying web-based social interactivity [6,30], or applying different BCTs in each arm [31,32]. The authors used a variety of interactive platforms to deliver interventions. The types of social media reported were distributed as follows: tailored, interactive websites (31 studies); Facebook only (23 studies); and a combination of tailored websites and other social media components (13 studies). Although not the focus of this review, self-directed strategies used in the intervention arms of the studies were noted. These included sharing informational and educational materials through means such as email [33-35] and video recordings [36,37]. Participants also had access to noninteractive aids such as pedometers [38,39] and used text messages [40,41] and personal diaries [42] to support their offline activities.

The included studies cover a variety of population groups, including college students [43], men who have sex with men [44], parents [45], patients with chronic diseases [46,47], and pregnant women [42,48].

We classified the outcomes of interest for the systematic review into 4 main categories: (1) health-related behaviors, (2) physical health, (3) well-being, and (4) psychological health. Health-related behaviors and physical health were the most reported outcomes and were identified in 40 and 25 studies, respectively. Health-related behaviors referred to the activities or practices taken by an individual, which either enhanced or deteriorated their health. It included measures such as physical activity [6,30,32,33,38,39,41,43,46,47,49-67], diet/nutrition [47,49,52,54,56,58,59,66,68,69], screening/testing [35,44,70-72], smoking [7,73-77], and medication adherence [45,78,79]. Physical health was used to describe any physiological measure such as BMI [32,41,56,58,63,67] and weight [38,40,48-50,52,59,65-67,69,80-84] but not biochemical markers of physiological health.

Well-being and psychological health were reported in 11 and 8 studies, respectively. Well-being was used to classify measures of quality of life [46,50,57,59,61,68,85], whereas psychological health was used to classify measures of psychological functioning, including depression [47,49,57,86,87], stress/distress [49,68,88], and anxiety [49,86].

### Capturing Behavior Change Techniques Unique to Social Media

As shown in the examples in Table 2, in identifying BCTs across studies, 3 nuanced operationalizations of existing BCTs emerged that did not fit neatly within the existing taxonomy specifications without losing content. Our new instructions allowed us to capture overt endorsement and virtual rewards noted in specific interventions [67,89]. Overt endorsement consists of open approval of a message by participants, and it can be observed in behaviors such as forwarding emails and comments, likes, and *retweets* on Facebook and Twitter [47,48]. Virtual rewards consist of a system that allows collecting prizes in the form of *smiles, congratulations, badges, virtual gifts, or stars* to encourage participants in their progress toward achieving their goals [47,48].

*Virtual rewards* (by design), although in principle consistent with the *rewards (behavior)* BCT, have a level of nuance within the web-based environment that we decided it was worth making an explicit distinction in coding instructions for that BCT. Namely, we coded all statements that described overt endorsement such as *likes, smiles, virtual gifts, Twitter posts,* and *public post on profile page wall* as *social support (unspecified)* when these statements came from participants themselves in a natural manner. However, we coded these same statements as *nonspecific reward* or *social reward* when they were embedded in the design of the intervention to acknowledge the achievement of participants.

**Table 2:** Application of newly created rules for capturing virtual rewards.

New instructions	Representative example of coded statements	Behavior change technique taxonomy version 1 code
Overt endorsement (from participants)	“Participants in this intervention were free to post their own relevant messages through videos, text, pictures and so on. They could engage in relevant discussions via commenting.” [90]	Social support (unspecified)
Virtual rewards (by design)	“It included gamification features, such as awards for individual and team step-logging and step-count achievements and the ability to send virtual gifts to teammates.” [61]	Social reward
Virtual rewards (by design)	“Engagement was rewarded in the intervention group with points, badges and gradual revealing of graphic-level images and other virtual elements.” [89]	Nonspecific reward

### Identification of Behavior Change Techniques in the Studies

We identified 46 out of 93 techniques from the BCTTv1 across the 71 included studies, which are displayed in Multimedia Appendix 3. BCTs were mainly reported in the Methods section of the studies (68 studies). We also identified BCTs from additional sources, such as protocols (11 studies), study appendices (4 studies), and author-identified companion papers (4 studies).

### Behavior Change Techniques Applied in Interactive Social Media and Self-Directed Components

More than 55% (26/46) of the BCTs identified were applied in the interactive social media component of interventions (two-way communication and interaction between and among participants). The top 15 BCTs applied across interactive social media and self-directed components of studies (one-way communication or no social interaction) are displayed in Table 3. As expected, *social support (unspecified)* was the most common BCT identified in interactive social media components (51 studies) [6-8,28,30,32,35,36,38,39,41,44-47,50-55,57,59,61, 63-66,69-73,75,77-80,82,83,85,87-95], followed by *instruction on* *how to perform the behavior* (21 studies) [28,36,37, 46-49,52,61,64,68,72,76,77,82,84,85,91,95-97], *credible source* (16 studies) [7,28,30,35,36,39,47,55,64,69,72,76,79,94,95,97], *social comparison* (8 studies) [36,43,48,54,61,65,80,82], and *information about health consequences* (8 studies) [28,43,64,68,72,83,90,95]. On the other hand, *instruction on how to perform the behavior* was the most commonly identified BCT in the self-directed component of interventions (39 studies) [6-8,30,32,34,38-41,43-45,50,51,53,55-60,62,65-67,69,71,74,75,79-81, 83,86,87,89,93,94], followed by *self-monitoring of behavior* (32 studies) [6,30,32,33,38-41,45,46,48,50,51,54-58,60,63-66, 69,76,79,81-84,92,98], *credible source* (29 studies) [8,32,34,38,41,44,45,48,50,53,56,58,59,62,65,66,68, 74,75,77,80-82,85,88,90,92,93,96], *goal-setting behavior* (25 studies) [6,30,32,38,39,41,48,50,51,54,55,57-60,65-67,74,75, 81-84,98], and *adding objects to the environment* (24 studies) [30,32,38-41,46,48-51,56-61,66,70,80,81,83,93,98].

**Table 3:** Top 15 behavior change techniques captured in the components of interventions (n=71).

Behavior change techniques	Social media, n^a^ (%)	Self-directed, n^a^ (%)
1.1 Goal setting (behavior)	7 (10)	25 (35)
1.2 Problem solving	6 (8)	13 (18)
1.4 Action planning	N/A^b^	19 (27)
2.2 Feedback on behavior	3 (4)	19 (27)
2.3 Self-monitoring of behavior	5 (7)	32 (45)
2.4 Self-monitoring of outcome(s) of behavior	2 (3)	N/A
3.1 Social support (unspecified)	51 (72)	12 (17)
3.2 Social support (practical)	7 (10)	17 (24)
3.3 Social support (emotional)	3 (4)	N/A
4.1 Instruction on how to perform the behavior	21 (30)	39 (55)
5.1 Information about health consequences	8 (11)	17 (24)
6.1 Demonstration of the behavior	7 (10)	11 (15)
6.2 Social comparison	8 (11)	N/A
7.1 Prompts/cues	7 (10)	21 (30)
8.7 Graded tasks	N/A	14 (20)
9.1 Credible source	16 (23)	29 (41)
11.1 Pharmacological support	N/A	9 (13)
12.2 Restructuring the social environment	5 (7)	N/A
12.5 Adding objects to the environment	N/A	24 (34)

^a^Behavior change techniques applied in interactive social media components and self-directed components.

^b^N/A: not applicable.

### Behavior Change Techniques Applied in the Intervention and Control Arms

The top 5 BCTs applied in intervention arms of the included studies: *social support (unspecified*; 56 studies) [6-8,28,30,32-36,38-41,44-47,52-62,64-66,69,70,72,73,75,77-83,85-95,97], *instruction on how to perform the behavior* (37 studies) [8,28,36,37,40,44-48,52,53,56-58,61,64,67,68,72,75,77,79,81-87,89,91,93-97], *credible source* (35 studies) [7,8,28,34-36,39,41,44,45,47, 48,53,56,58,59,62,64,65,68,69,72,75,77,79,81,82,85,88,92-97], *self-monitoring of behavior* (25 studies) [6,32,39,40,45-48, 56-58,61,64,65,69,76,78-84,88,92], and *prompts/cues* (24 studies) [33,39-41,43,47,48,52,55,56,58,61,64,65,69,73,78,79, 81,82,89,92,93,97]. The content of the control arms was generally reported as *usual care*. Only 9 different BCTs were uniquely applied in the control arms of the studies. *Instruction on how to perform the behavior*, *social support (unspecified)*, and *information about health consequences* constituted the most commonly applied BCTs in control arms only and were identified in 8 [7,29,34,38,39,41,59,66], 3 [50,51,63], and 2 [38,63] studies, respectively. The top 15 BCTs applied to the intervention and control arms of the included studies are displayed in Table 4.

Across the included studies, 14 of the top 15 BCTs applied in intervention arms only were also used in studies with BCTs applied in both arms. The exception was *social comparison*, which was solely identified in the intervention arms of 11 studies [32,33,36,43,48,54,58,61,65,80,82]. As shown in Table 4, the ranking of BCTs also changed with the simultaneous application of BCTs in both study arms in comparison with application in intervention arms only.

**Table 4:** Top 15 behavior change techniques in the study arms (n=71).

Behavior change techniques	Intervention, n^a^ (%)	Control, n^a^ (%)	Intervention and control, n^a^ (%)
1.1 Goal setting (behavior)	19 (27)	N/A^b^	13 (18)
1.2 Problem solving	13 (18)	1 (1)	5 (7)
1.4 Action planning	14 (20)	1 (1)	6 (8)
2.2 Feedback on behavior	16 (23)	N/A	6 (8)
2.3 Self-monitoring of behavior	25 (35)	1 (1)	11 (15)
3.1 Social support (unspecified)	56 (79)	3 (4)	4 (6)
3.2 Social support (practical)	18 (25)	N/A	6 (8)
4.1 Instruction on how to perform the behavior	37 (52)	8 (11)	15 (21)
5.1 Information about health consequences	15 (21)	2 (3)	8 (11)
6.1 Demonstration of the behavior	14 (20)	N/A	4 (6)
6.2 Social comparison	11 (25)	N/A	N/A
7.1 Prompts/cues	21 (30)	N/A	7 (10)
8.7 Graded tasks	9 (13)	N/A	5 (7)
9.1 Credible source	35 (49)	1 (1)	9 (13)
11.1 Pharmacological support	N/A	N/A	4 (6)
11.3 Conserving mental resources	N/A	1 (1)	N/A
12.5 Adding objects to the environment	13 (18)	N/A	11 (15)
13.2 Framing/reframing	N/A	1 (1)	N/A

^a^Behavior change techniques applied in intervention arms only, control arms, and intervention and control arms simultaneously.

^b^N/A: not applicable.

### Behavior Change Techniques Applied to Group and Individual Participants

*Social support (unspecified)* was the most commonly applied BCTs to a group of participants (50 studies) [6-8,30,32,33,36,38,39,41,44-46,50-61,63-66,69-73,75-78,80,82,83,85-87,90-95]. It was then followed by *instruction on how to perform the behavior* (24 studies) [36,37,44,45,48,52,53,55-58, 62,64,67,72,76,82,84,85,87,93,95-97], *credible source* (23 studies) [7,30,35,36,39,44,45,47,53,55,56,58,64,69,72,75,77, 79,85,90,94,95,97], *demonstration of the behavior* (12 studies) [6,36,52,53,55,56,58,62,64,76,93,94], and *social support practical* (11 studies) [7,32,33,39,48,52,53,58,68,72,76]. When BCTs were applied to individuals, *instruction on how to perform the behavior* was the most frequent (36 studies) [6-8,28,30,32,34,38-41,43,46,47,49-51,59-61,65,66,68,69,71,74,75,77,79-81, 83,86,89,91,94], followed by *self-monitoring of behavior* (34 studies) [6,30,32,33,38-41,46-48,50,51,54-58,60,61,63,65,66, 69,76,78-83,88,92,98], *goal-setting behavior* (30 studies) [6,30,32,38,39,41,46-48,50,51,54,55,57-60,65-67,74-77, 81-84,86,98], *adding objects to the environment* (24 studies) [30,32,38-41,46,48-51,56-61,66,70,80,81,83,93,98], *and credible source* (22 studies) [8,28,32,34,38,41,48,50,59, 62,65,66,68,74,77,80-82,88,92,93,96]. Table 5 shows the most frequent BCTs applied to group and individual participants.

**Table 5:** Top 15 behavior change techniques in participant settings (n=71).

Behavior change techniques	Group setting, n^a^ (%)	Individual setting, n^a^ (%)
1.1 Goal setting (behavior)	N/A^b^	30 (42)
1.2 Problem solving	5 (7)	14 (20)
1.4 Action planning	5 (7)	16 (23)
1.5 Review behavior goal(s)	N/A	10 (14)
2.2 Feedback on behavior	N/A	19 (27)
2.3 Self-monitoring of behavior	3 (4)	34 (48)
2.8 Feedback on outcome(s) of behavior	N/A	7 (10)
3.1 Social support (unspecified)	50 (70)	13 (18)
3.2 Social support (practical)	11 (15)	13 (18)
3.3 Social support (emotional)	3 (4)	N/A
4.1 Instruction on how to perform the behavior	24 (34)	36 (51)
5.1 Information about health consequences	6 (8)	19 (27)
5.3 Information about social and environmental consequences	3 (4)	N/A
6.1 Demonstration of the behavior	12 (17)	N/A
6.2 Social comparison	9 (13)	N/A
7.1 Prompts/cues	9 (13)	19 (27)
8.1 Behavioral practice/rehearsal	4 (6)	N/A
8.7 Graded tasks	N/A	13 (18)
9.1 Credible source	23 (32)	22 (31)
12.2 Restructuring the social environment	6 (8)	N/A
12.5 Adding objects to the environment	N/A	24 (34)

^a^Behavior change techniques applied to individuals in the group setting and individual participants.

### Low- and High-Intensity Use of Behavior Change Techniques

We characterized the intensity of delivering BCTs by identifying the modalities used to expose participants to a BCT. BCTs that were delivered using at least two modalities were coded as high intensity. Participants were exposed to BCTs through interactive social media and self-directed components in intervention or control arms of the studies and in a group or individually. The top 5 BCTs that were delivered with high intensity included i*nstruction on how to perform the behavior* (35 studies) [7,28,32,34,36-38,40,45,48,49,52,53,55-59,62,65-69,72,74,75,77,79,80,83, 85,93,95,96], social *support (unspecified*; 28 studies) [7,8,28,32-35,37,40,41,45,50,51,56,61-67,71,73-75,77,81,86], s*elf-monitoring of behavior* (16 studies) [32,50,51,54,57,58,60,63,64,66,67,69,78,82,92,96], *information about health consequences* (13 studies) [7,32,34,36,38,48,50,56,59,80,81,95,96], and *credible source* (13 studies) [7,32,34,36,38,48,50,56,59,80,81,95,96]. The top 15 most frequent BCTs coded for high-intensity delivery are displayed in Table 6.

**Table 6:** Top 15 behavior change techniques coded for high-intensity delivery (n=71).

Behavior change techniques	Value, n (%)
1.1 Goal setting (behavior)	10 (14)
1.2 Problem solving	5 (7)
1.4 Action planning	8 (11)
1.5 Review behavior goal(s)	3 (4)
12.5 Adding objects to the environment	8 (11)
2.2 Feedback on behavior	8 (11)
2.3 Self-monitoring of behavior	16 (23)
3.1 Social support (unspecified)	28 (39)
3.2 Social support (practical)	4 (6)
4.1 Instruction on how to perform the behavior	35 (49)
5.1 Information about health consequences	15 (21)
6.1 Demonstration of the behavior	6 (8)
6.2 Social comparison	3 (4)
7.1 Prompts/cues	3 (4)
9.1 Credible source	13 (18)

## Discussion

### Principal Findings

We found that the BCTTv1 was applicable to studies promoting health behaviors using social media in a web-based environment. Given that all these studies focused on interactive web-based platforms, it is surprising that relatively few studies have reported leveraging the full spectrum of social comparison and social support BCTs. Furthermore, few studies have reported using BCTs that have been associated with effective behavior change in other settings (eg, goal setting and action planning) [99,100].

We made 3 adaptations to the coding structure to accurately and specifically code overt endorsement and virtual rewards, which we decided to be a type of social support when provided by peers through the internet and a type of social reward when provided by intervention designers. We also developed a method for coding the intensity of BCTs, which allowed us to identify studies with more intense use of BCTs. We added to the description of some BCTs to capture these nuances of interactive social media. Kadushin [101] and Southwell [102] highlighted the potential of overt endorsement and virtual rewards in influencing individual attitudes and health-related behaviors. We also noted that these techniques were reported in other studies as a measurement of engagement with participants and reach of interventions [67,90].

The interventions were mainly delivered through tailored, interactive platforms (ie, platforms developed by the research team ). At the time of conducting this research, Facebook remained the most commonly used platform among the mainstream social media outlets for intervention studies and was used either alone or in combination with other social media components. Nonetheless, this does not dismiss the popularity of other social media platforms implemented in other countries. Social media platforms usually allow social interactions beyond geographical boundaries. We noted that none of the included studies had a global focus, that is, all were conducted within a specific country or region. In addition, with the widespread digital technologies, we expected to see more than one study from low- and middle-income countries. Resource settings [103] and research capacity [104,105] might be explored when assessing possible reasons for limited implementation internationally.

Almost all the studies used a variety of BCTs to deliver interventions through social media. The identified BCTs were applied in both the intervention and control arms of the studies. For this reason, it is essential to identify the intensity with which BCTs are applied. Identifying the intensity can help to determine if the extent of exposure makes a difference in the impact of changing behavior. Even after considering intensity, caution should still be applied when assessing the role of BCTs in changing the behavior of participants because some BCTs were applied with almost equal intensity in the control and intervention arms. This was observed for the BCT *instruction on how to perform the behavior.*

In addition, the content of control arms was mainly reported as *usual care*, with often no accompanying details on the features of such conditions. This situation makes it difficult to adequately characterize the content of control arms and warrants more analysis in attributing effectiveness to BCTs applied in intervention arms [52]. The types of BCTs applied to individuals in a group setting and those applied to individual participants varied greatly. This provides opportunities to explore which BCTs are optimal in a group setting and to identify which ones are inherently appropriate for individual activity only. Additional research can help elucidate the mechanisms of action of BCTs in interventions and further contribute to their optimal use to foster behavior change.

### Limitations

One limitation of this research is that we relied on what was reported in the source studies. We attempted to check the web-based sites, but a lot had changed since the publication of the studies in the review. Therefore, the relative paucity of some BCTs (eg, social comparison and goal setting) may be related to insufficient detail in the published papers to code these aspects of the intervention. We encourage future reports of interventions to provide screenshots or access to the version of the platform used at the time of the intervention as additional files or links. In addition, the use of validated tools such as the Consolidated Standards of Reporting Trials eHealth can help improve the description and reporting of web-based interventions, thereby facilitating their implementation and replication to improve health outcomes [106].

Future research can also contribute to better characterize BCTs applied to foster social support in interactive social media interventions. *Social support (unspecified)* was the most commonly applied BCT in the studies analyzed. The definition of this BCT is comprehensive enough to allow the identification of social assistance with no specific descriptors, whereas BCTs such as *social support (practical)* and *social support (emotional)* allow capturing what social assistance entails. A better characterization of *social support* in interventions could help to distill in what ways this particular social interaction makes a difference in the behavior of participants.

### Comparisons With Prior Work

A recent review that focused on social media features in web-based interventions reported that communication in the form of forums is the most common social media feature for sustaining behavior change [15]. These results are aligned with those of this SWAR, which shows that generic social support is the most common BCT used in interactive social media interventions, which was mainly captured in interventions that use discussion forums, group activities, and other exchanges to foster mutual support among participants.

### Future Work

In this study, we provide an overview of the most commonly reported BCTs applied in social media intervention studies. As social media interventions continue to increase in popularity, it may be useful in future work to assess how the presence of particular BCTs or a combination of BCTs is associated with effects on health behavior change.

### Conclusions

Our newly developed coding instructions along with BCTTv1 were useful in characterizing behavior change content in social media interventions and evaluating its effectiveness. This assessment of BCTs identified nuances in the operationalization of the BCTTv1 to characterize the unique features of interactive social media interventions for health promotion. These characteristics may be considered when designing social media platforms aimed at promoting public health. Our coding could further help deconstruct the complexity of interventions that use digital technologies and provide further understanding of the role of specific BCTs on behavior change. Although other studies highlighted the effectiveness of BCTs, such as goal setting and action planning, very few of the included studies used them. Increasing the use of BCTs is known to be effective in improving social media interventions.

## Appendix

Multimedia Appendix 1 User manual for coding behavior change techniques using the Behavior Change Technique Taxonomy version 1 in social media–based health interventions.

Multimedia Appendix 2 Sample of search strategy and results from the Medical Literature Analysis and Retrieval System Online, or MEDLARS Online database.

Multimedia Appendix 3 Distribution of behavior changing techniques captured across the 71 included studies.

## Abbreviations

BCT: behavior change technique
BCTTv1: Behavior Change Technique Taxonomy version 1
MEDLINE: Medical Literature Analysis and Retrieval System Online, or MEDLARS Online
RCT: randomized controlled trials
SWAR: study within a review
WHO: World Health Organization
