# Supplementary material for: Behavior Change Techniques Included in Reports of Social Media Interventions for Promoting Health Behaviors in Adults: Content Analysis Within a Systematic Review
Source: J Med Internet Res. 2020 Jun 11;22(6):e16002. doi: 10.2196/16002 (PMC7317628; doi:10.2196/16002)
Supplement: Multimedia Appendix 1 [file jmir_v22i6e16002_app1.docx]

# Appendix A: Sample of search strategy

**Database: Ovid MEDLINE(R) Epub Ahead of Print, In‐Process & Other Non‐Indexed Citations, Ovid MEDLINE(R) Daily and Ovid MEDLINE(R) <1946 to Present>**

**Search Strategy:**

**‐‐‐‐‐‐‐‐‐‐‐‐‐‐‐‐‐‐‐‐‐‐‐‐‐‐‐‐‐‐‐‐‐‐‐‐‐‐‐‐‐‐‐‐‐‐‐‐‐‐‐‐‐‐‐‐‐‐‐‐‐‐‐‐‐‐‐‐‐‐‐‐‐‐‐‐‐‐‐‐**

1 exp Social Media/ (4579)

2 Blogging*.mp. [mp=title, abstract, original title, name of substance word, subject heading word, keyword heading word, protocol supplementary concept word, rare disease supplementary concept word, unique identifier, synonyms] (1004)

3 Blogging/ (870)

4 Communications Media/ (1458)

5 Social Networking/ (2110)

6 (social adj2 media).tw. (5844)

7 ((virtual or online) adj2 (communit$ or network$)).tw. (2204)

8 "Web 2.0".tw. (558)

9 Facebook.tw. (2224)

10 Twitter.tw. (1876)

11 MySpace.tw. (79)

12 Tumblr.tw. (34)

13 instagram.tw. (143)

14 pinterest.tw. (37)

15 wiki$.tw. (1176)

16 YouTube.tw. (1110)

17 vimeo.tw. (5)

18 Flickr.tw. (82)

19 Delicious.tw. (680)

20 blog$.tw. (1490)

21 (linkedin or linked in).tw. (4446)

22 (sixdegrees or six degrees).tw. (766)

23 weibo.tw. (55)

24 curediva.tw. (0)

25 connectedliving.tw. (0)

26 patientslikeme.tw. (47)

27 wego.tw. (7)

28 caringbridge.tw. (2)

29 crowd sourc$.tw. (246)

30 crowdsourc$.tw. (735)

31 hash tag$.tw. (3)

32 hashtag$.tw. (179)

33 microblog$.tw. (164)

34 push technolog$.tw. (18)

35 facetime$.tw. (41)

36 Friendster.tw. (5)

37 Gchat.tw. (1)

38 g‐chat.tw. (0)

39 google maps.tw. (152)

40 Kik.tw. (49)

41 reddit$.tw. (56)

42 subreddit$.tw. (4)

43 snapchat$.tw. (21)

44 tweet$.tw. (1434)

45 wechat$.tw. (33)

46 whatsapp$.tw. (144)

47 MXit.tw. (6)

48 QQ.tw. (797)

49 Qzone.tw. (0)

50 baidu.tw. (88)

51 viber.tw. (6)

52 Vkontakte.tw. (0)

53 Odnoklassniki.tw. (0)

54 Facenama.tw. (0)

55 (YY and (social adj2 network$)).tw. (0)

56 (QQ and (social adj2 network$)).tw. (2)

57 (vine and (social adj2 network$)).tw. (0)

58 (LINE and (social adj2 network$)).tw. (173)

59 or/1‐58 (25532)

60 limit 59 to yr="2000‐ 2016" (18857)

61 (pre‐intervention$ or preintervention$ or pre intervention$ or post‐intervention$ or postintervention$ or post intervention$).ti,ab. (21795)

62 demonstration project$.ti,ab. (2546)

63 (pre‐post or pre test$ or pretest$ or posttest$ or post test$ or (pre adj5 post)).ti,ab. (107401)

64 trial.ti. or ((study adj3 aim$) or our study).ab. (1238159)

65 (before adj10 (after or during)).ti,ab. (483594)

66 (quasi‐experiment$ or quasiexperiment$ or quasi random$ or quasirandom$ or quasi control$ or quasicontrol$ or ((quasi$ or experimental) adj3 (method$ or study or trial or design$))).ti,ab,hw. (142022)

67 (time points adj3 (over or multiple or three or four or five or six or seven or eight or nine or ten or eleven or twelve or month$ or hour$ or day$ or more than)).ab. (16023)

68 (time series adj2 interrupt$).ti,ab,hw. (2307)

69 pilot.ti. (63173)

70 Pilot projects/ (115494)

71 (clinical trial or controlled clinical trial or multicenter study or randomized controlled trial or pragmatic clinical trial).pt. (1009144)

72 (multicentre or multicenter or multi‐centre or multi‐center).ti. (47610)

73 random$.ti,ab. or controlled.ti. (1118638)

74 (control adj3 (area or cohort$ or compare$ or condition or design or group$ or intervention$ or participant$ or study)).ab. (692017)

75 evaluation studies as topic/ or prospective studies/ or retrospective studies/ or non‐randomized controlled trials as topic/ or interrupted time series analysis/ or controlled before‐after studies/ (1366715)

76 (during adj5 period).ti,ab. (399506)

77 ((strategy or strategies) adj2 (improv$ or education$)).ti,ab. (31325)

78 (rat or rats or cow or cows or chicken$ or horse or horses or mice or mouse or bovine or animal$).ti. (1642704)

79 exp animals/ not humans.sh. (4806730)

80 (or/61‐77) not (or/78‐79) (4178077)

81 60 and 80 (3367)
