# Supplementary material for: Behavior Change Techniques Included in Reports of Social Media Interventions for Promoting Health Behaviors in Adults: Content Analysis Within a Systematic Review
Source: J Med Internet Res. 2020 Jun 11;22(6):e16002. doi: 10.2196/16002 (PMC7317628; doi:10.2196/16002)
Supplement: Multimedia Appendix 2 [file jmir_v22i6e16002_app2.docx]

**User Manual for Coding Behaviour Change Techniques (BCTs) in Social Media Based Health Interventions**

| **Coding overview** |
| --- |

All 93 BCTs are listed in columns across the top row of the coding tool. Each BCT category is supported by 4 columns (statement, section, setting, study arm) which capture the context of the BCT. Any columns that do not relate to the study being considered are to be left blank.

**Table 1: Data extraction instructions category**

| **Study arm description** | Specify the number of arms and the components of each arm (intervention or control) as described by the author. |
| --- | --- |
| **BCT** | Enter either ‘I’ or ‘G’ in this column. This indicates that a) this BCT is present and b) that it was applied in either an **I**ndividual or **G**roup setting. |
| **Statement** | Copy and paste the text that indicates the presence of a BCT and any additional contextual information. Some sentences can indicate multiple BCTs so coders should bold the portion of the text that relates to the BCT being coded. |
| **Section** | Indicate the section of the article where the BCT was found.  (background, methods, etc.) |
| **Setting** | Enter ‘**Y**’ into this column if the BCT was applied in an interactive social media setting, **N** if it was not applied in an interactive social media setting |
| **Study arm** | Enter ‘intervention’ or ‘control’; OR ‘intervention; control’ (if both) to indicate which study arm the BCT was applied to. If there are more than 2 arms, indicate which arm the BCT was applied to and the name of the arm as defined by the author (e.g. intervention - group D; intervention - group E). |

**Example:**

| **Study arms description** | **4.1. Instruction on how to**  **perform the behavior** | Statement | Section | Setting - Social media or other (Y or blank) | Study Arm - Intervention or Control |
| --- | --- | --- | --- | --- | --- |
| **Intervention and contro**l; " The **intervention** group  had an online social network (Instagram) accounts;  **control** group had no such account.” | I | **they received the**  **exercise program video link** (can be accessed on YouTube  https://www.youtube.com/watch?v=fcN37TxBE s) | Intervention | Y | Intervention; Control |

| **Instructions for Data Extraction by Category** |
| --- |

**I. Study Arm Description:**

Specify the number of arms and the components of each arm (intervention or control) as described by the author. When there are multiple intervention arms, record author’s classification for each arm. See Study Arm section below for details about BCT data extraction.

Example from a 6 arm study: “**LF**= lecture and FB for less than 15 min/wk; **LF15**= lecture and FB for more than 15 mins/wk; **L** = lecture; **F**=FB less than 15 min/wk; **F15**=FB for more than 15 mins/wk; **C** = control, no lecture/FB”

**II. BCT:**

Enter either ‘I’, ‘G’, or ‘N/A’ into this column. This indicates that a) this BCT is present and b) that it was applied in either an **I**ndividual or **G**roup setting.

For information about when to use N/A, see “IV. Section” instructions listed below.

# **Coding Decisions**

We do not want to under represent the presence of BCTs but also need to be cautious of inferences when there is not enough information provided. Principally, coders should always use the BCT Taxonomy v1 as a guiding rule.

However, when a coder presumes that a BCT is present but it is either a) not reported in accordance to the BCT Taxonomy v1, or b) there is not enough context provided in the original article, coders can base their judgement on several factors.

## **A)** **Explicit Reporting of Behaviour Change**

If the article explicitly reports utilizing behaviour change techniques/strategies/theories, coders can code the BCTs mentioned in the article even if they are not reported in accordance to the Taxonomy v1 as long as appropriate language is used

- E.g. “Participants received daily text messages tailored to each weight-related strategy that were based on effective behavior change techniques (e.g., recognizing cues, role modeling, reward)” (Herring, 2014, p. 4).
  - Because the article explicitly states using “behavior change techniques” and references specific techniques in the Taxonomy v1, coders would code:
    - “recognizing cues” → 7.1 Prompt/Cues
    - “role modeling” → 13.1 Identify self as a role model
    - “reward” → 10.10 Reward (outcome)
- Reminder from Taxonomy v1: If the BCT type (behaviour vs. outcome) is unspecified, coders are instructed to code the outcome type because if one were evaluating BCT effectiveness the outcome version is less likely to overestimate BCT effectiveness

**B) Recording multiple Doses**

If there are multiple doses of the same BCT within an intervention, add new rows for that study and capture the additional doses on separate rows.

Dose is defined as participants’ exposure to a BCT. Use additional rows correspondingly to the number of doses to be recorded.

- **Record multiple doses when:**

1. One BCT is applied more than once to influence the same behaviour
   - e.g. Intervention aims to improve healthy eating: “Participants in the intervention arm were provided with best practices guidelines on eating healthy and were instructed to watch short cooking videos on youtube”.
     1. These are both BCT 4.1 Instruction on how to perform the behaviour applied using two different modalities. Use additional rows to record the 2 doses.
2. One BCT is applied to influence multiple behaviours.
   - e.g. Intervention aims to help prevent unhealthy weight gain: “In addition to knowledge-based information relevant to the focus behaviors, the course included self-monitoring of eating, activity, sleep, and time management and goal setting related to changes in those behaviors.”
     1. Because the BCTs ( self-monitoring and goal setting) targets each 4 different behaviours- eating, activity, sleep, and time management- code these as 4 separate doses (adding a new row for each) for BCTs 2.3 Self-Monitoring of Behaviour AND 1.1 Goal Setting (Behaviour), respectively.
     2. Coders should bold the portion of the text related to the dose considered in the statement column. See Laska 2016 in the coding sheet for more examples.
     3. Note: if the article stated “Participants were instructed to record their daily meals as well as daily sugary beverage consumption”, **only code ONE dose** as these are both related to self-monitoring of eating behaviour. However, if the author did not bundle these behaviours as “eating” then code each behaviour as a separate dose.
   - Reminder from BCT Taxonomy v1: “Only code BCTs that are directly applied to the target behaviour(s) and population(s)”
3. One BCT is applied to more than one study arms (co-interventions or control) using one or multiple modalities.

**Example:**

| **4.1. Instruction on how to**  **perform the behavior** | Statement | Section | Setting - Social media or other (Y or N) | Study Arm - Intervention or Control |
| --- | --- | --- | --- | --- |
| I | **they received the exercise program video link** (can be accessed on YouTube  https://www.youtube.com/watch?v=fcN37TxBE s) | Methods | Nx | Intervention; Control |

E.g.

| **4.1. Instruction on how to**  **perform the behavior** | Statement | Section | Setting - Social media or other (Y or N) | Study Arm - Intervention or Control |
| --- | --- | --- | --- | --- |
| I | Participants were **provided a handbook which contents included how to manage the nutrition and eating problems during cancer symptoms.** | Methods | Nx | Control |
| I | Participants were **provided a handbook which contents included how to manage the nutrition and eating problems during cancer symptoms…** Additional: all components of the control group were delivered to the intervention group but through the group facebook page rather than face-to-face. | Methods | **Y** | intervention |

- **Interpretation of applied doses**

**Table 2: Summary table for recording multiple doses and high intensity use of BTCs in interventions**

| Dose | Intensity |
| --- | --- |
| 1 dose (1 unit) = 1 BCT and 1 Behaviour | One dose equals low intensity delivery |
| Multiple doses = 1 BCT and Multiple behaviours | More than one dose equals high intensity delivery |
| Multiple doses = Multiple BCTs and 1 behaviour |  |
|  |  |

## C) **BCT Speculation**

In cases where coders speculate that a BCT is present but the article does not explicitly report utilizing behaviour change techniques nor is the BCT reported in accordance with the BCT Taxonomy v1 (i.e. absence of an ‘action verb’), coders must ensure that enough context is available to base their judgement on and copy and paste any contextual information relating to the BCT to the coding tool in the ‘Statement’ column.

- E.g. “The purpose of this study was to test the effectiveness of a six-week Web-based multiple health behavior change program for adult survivors...The Post Office component was used so that participants could message each other individually for support.”
  - Coders can code BCT ‘3.1 Social Support (unspecified)’ because there is sufficient information to infer the presence of this BCT despite the absence of an action verb (‘given’, ‘prompted’, ‘asked’ etc.).

## **D) Additional Resources**

Coders are restricted to using the additional resources identified below to inform BCT coding. Coders will not be analyzing the social media platforms used in interventions to inform BCT coding. Social media platforms evolve quickly making it difficult to determine if all of the current features of the platform were available at the time of intervention. Furthermore, restricting BCT coding to the primary paper and the additional resources listed below permits replication.

If an additional resource is used, include the resource citation (“type of resource: Author, year”) in the ‘notes’ column. E.g. “used Protocol: Coffeng, 2012”

1. **Companion Papers:** Companion papers linked to the primary study in Covidence or companions identified by authors in the primary study can be used to inform BCT coding.
2. **Protocols:** BCTs found within study protocols can be coded. However, be cautious of any information that may have changed from the protocol to primary publication.
   1. E.g. Authors may have changed the names of the study arms between protocol and primary reporting. To be consistent, use classification from the primary study.
3. **Supplementary Documents:** supplementary documents found in the Appendix section of the primary study can be used to inform BCT coding.
   1. E.g. “Participants were provided with handbooks. See Suppl. File. 1 for more details.” When you open Suppl. File 1 and find that it is a page from the handbook that provides instructions on how to perform a stretch and the target behaviour is to increase physical activity, code ‘4.1 Instruction on how to perform the behaviour’.
4. **Journal Articles:** If the additional resource is a journal article for a separate intervention that provides more context about the program/tool used in the primary study, coders can code BCTs found in a referenced article as long as they relate directly to the included study.
   1. E.g. If the author directs readers to an additional journal article for details about the QuitNet program that was used in their intervention, coders can use the additional journal article’s information explicitly related to the QuitNet program to inform BCT coding.
5. **Handbook/Guidelines etc:** If the authors refer readers directly to a handbook or guidelines for additional information, coders should only code BCTs related to ‘informing’ participants unless there is sufficient evidence that participants followed all activities stated in the handbook/guidelines. Coders must also ensure that they are coding from the version used at the time of the intervention rather than the most recent version available (see article’s references).
   1. E.g. Jane 2017 “The Control Group (CG) were instructed to follow the Australian Government dietary guidelines [32] as well as the National Physical Activity Guidelines for Adults [33] as standard care.” No other information is provided in the article/companions/protocol about control group activities.
      1. When reading both guidelines it is evident that participants were provided with 5.1 information about health consequences and 4.1 Instruction on how to perform the behavior.
      2. There is inference of 8.2 Behaviour substitution within these guidelines ( e.g. “alternate your alcoholic drinks with mineral water or a diet soft drink. Use vegetables rather than biscuits for dipping”). However, participants were only instructed to follow the guidelines, there is no evidence that behaviour substitution was an explicit intervention component so coders cannot code this BCT.
         - Coders must be cautious of inferences and ensure that there is sufficient information available to inform BCT coding.

**III. Statement**

Copy and paste the text that indicates the presence of a BCT and any additional contextual information. Some sentences can indicate multiple BCTs so coders should bold the portion of the text that relates to the BCT being coded. If contextual information was copied from an additional resource, indicate this in the statement section.

Example:

| **4.1. Instruction on how to**  **perform the behavior** | Statement | Section | Setting - Social media or other (Y or N) | Study Arm - Intervention or Control |
| --- | --- | --- | --- | --- |
| I | **instructed to follow the Total Wellbeing Diet** developed by the Commonwealth Scientific and Industrial Research Organisation, following rigorous scientific testing and proven to result in weight loss;. Supplement 2: **includes healthy recipes** | Methods | Nx | Intervention FG |

## **IV. Section**

Behaviour Change Techniques (BCTs) are often reported in the methods/design/intervention section of the article.

1. If a BCT is mentioned in the abstract section and then reiterated in the methods/design/intervention section, users should extract only the information in the methods section unless there is information in the abstract that wasn’t reiterated.
2. If the entire statement was copied from an additional resource, indicate what section of the resource the statement was found and use parentheses to indicate what resource was used.
   - Reminder: record the additional resources used for coding in the ‘notes’ column.

**Example:**

| **1.4. Action planning** | Statement | Section | Setting - Social media or other (Y or N) | Study Arm - Intervention or Control |
| --- | --- | --- | --- | --- |
| G | GMI-session included: worksheets to **help extract planning goals (when, where, with whom?)** for improving physical activity, and rewards for favourable outcomes are formulated. | methods (protocol) | Nx | Intervention (group 1); Intervention (group 2) |

1. If coders find a BCT in the ‘results’ section without it being referenced in the intervention section, coders cannot infer the presence of a BCT; however, we still want to record these instances in the coding sheet.
   - E.g. There is no evidence in the intervention section to support the use of goal setting, but the results section states “as a result of goal setting, 5% of participants in the intervention group quit smoking within six months” Because goal setting was not mentioned as a component of the intervention, it should not be coded.
   - Coders must still record this in the sheet the same way all other BCTs are recorded, but must enter ‘N/A’ into the 1.3 Goal Setting (outcome) category instead of either ‘I’ or ‘G’.

**Example:**

| **1.3. Goal setting (outcome)** | Statement | Section | Setting - Social Media or other (Y or N) | Study Arm – Intervention or Control |
| --- | --- | --- | --- | --- |
| N/A | as a result of goal setting, 20% of participants in the intervention group quit smoking within three months | Outcomes | Nx | Intervention |

**V. Setting**

In order to code a BCT setting as ‘social media’, the intervention must use the interactive component of the social media platform. If not enough information is provided, the BCT cannot be considered to be applied in an interactive social media setting so coders must leave this cell blank.

E.g. El-Aisa 2016 is an intervention to increase physical activity. Instagram is used as the interactive component of the intervention; however, participants are also instructed to watch an exercise video on YouTube. Based on the available information, the YouTube video is solely used as an educational resource and does not utilizes the multi-way communication functionality of the platform. Therefore, the setting of the BCT related to the YouTube video should be left blank.

- Reminder from the protocol**:** one-way messages and posts or direct contact with a health care provider are not classified as interactive social media. If the intervention does not utilize social media with multi-way communication, it is excluded. Therefore, BCTs don’t have to be interactive, but to be classified as a social media setting, there must be multi-way communication.

**VI. Study Arm**

Enter ‘intervention’ or ‘control’; OR ‘intervention; control’ (if both) to indicate which study arm the BCT was applied to. If there are more than 2 arms, indicate which arm(s) the BCT was applied to and the name of the arm(s) as defined by the author [e.g. “intervention (group D); Intervention (Group E); Control (Group A)”]. See Bramlet Mayer 2012 in the coding sheet for more examples.

| **Additional Rules** |
| --- |

1. **Coding virtual rewards:**

There is currently not a BCT that adequately captures virtual rewards. Therefore, statements describing virtual rewards such as “likes” “smiles” “virtual gifts” “ public post on profile page wall” “Twitter posts.

- 1. When virtual rewards or endorsement are initiated by or coming from intervention participants code:
     1. 3.1“social support unspecified”
  2. When virtual rewards are initiated by intervention designers code
     1. 10.3 Non-specific reward’ unless the author explicitly states that virtual rewards can be exchanged for material goods.
     2. If the virtual reward is in the form of praise or encouragement (e.g. “congratulations!”) then code ‘10.4 social reward’.
