# Supplementary figures and images for: Behavior Change Techniques Included in Reports of Social Media Interventions for Promoting Health Behaviors in Adults: Content Analysis Within a Systematic Review
Source: J Med Internet Res. 2020 Jun 11;22(6):e16002. doi: 10.2196/16002 (PMC7317628; doi:10.2196/16002)

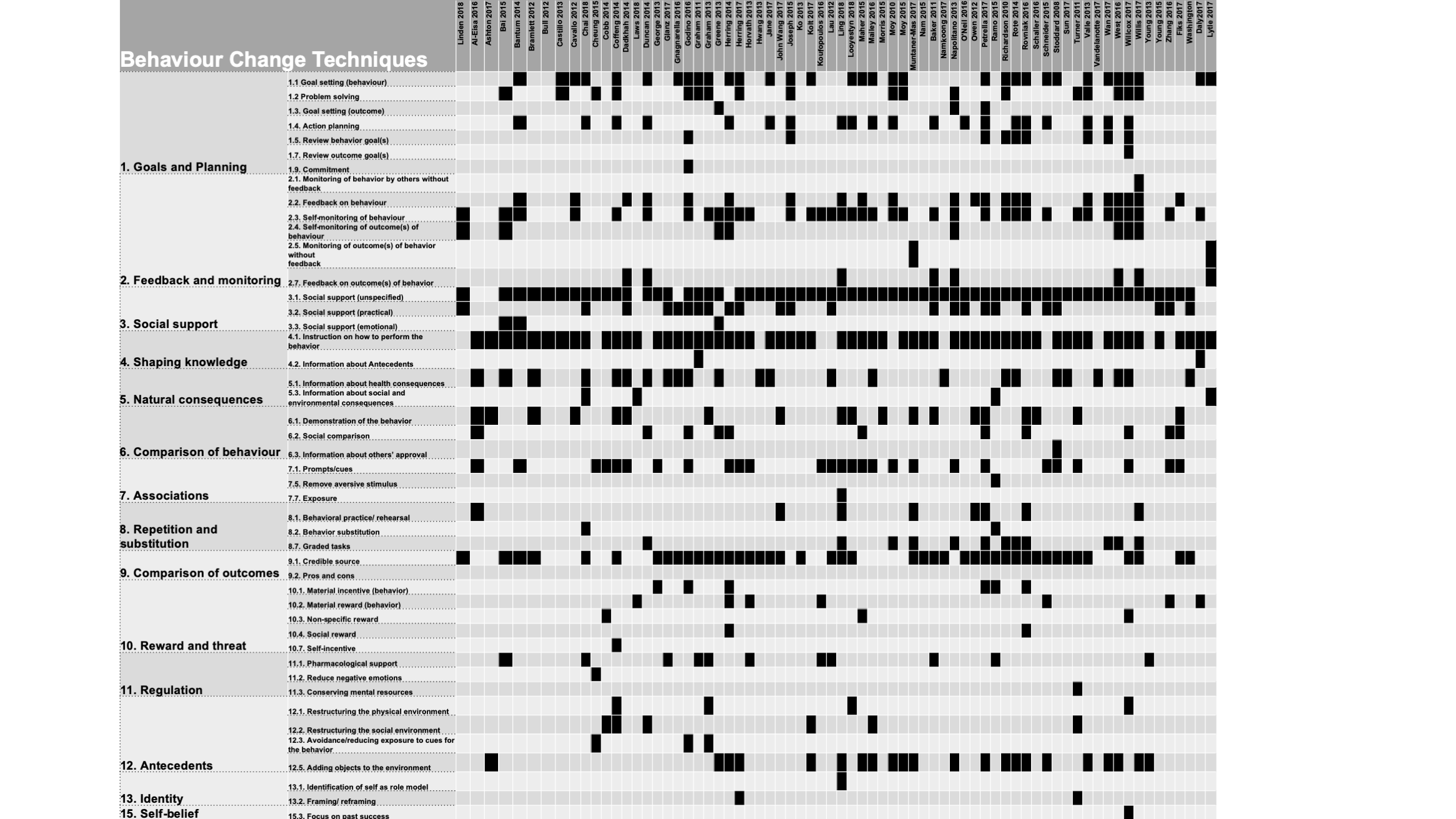

Supplement: Multimedia Appendix 3 [file jmir_v22i6e16002_app3.png]
